# Changes in imatinib plasma trough level during long-term treatment in patients with intermediate- or high-risk gastrointestinal stromal tumors: Relationship between covariates and imatinib plasma trough level

**DOI:** 10.3389/fsurg.2023.1115141

**Published:** 2023-02-23

**Authors:** Xingye Wu, Yinggang Ge, Xuemei He, Juan Li, Jun Zhang

**Affiliations:** ^1^ Department of Gastrointestinal Surgery, The First Affiliated Hospital of Chongqing Medical University, Chongqing, China; ^2^ Department of Ultrasound, The First Affiliated Hospital of Chongqing Medical University, Chongqing, China; ^3^ Department of Pharmacy, The First Affiliated Hospital of Chongqing Medical University, Chongqing, China

**Keywords:** gastrointestinal stromal tumors, imatinib, adjuvant therapy, therapeutic drug monitoring, plasma concentration

## Abstract

**Background:**

Imatinib is the first-line adjuvant treatment for gastrointestinal stromal tumors (GISTs). Considering that some studies have suggested that imatinib (IM) plasma trough levels (C_min_) change with time, the aim of this study is to assess the changes in IM C_min_ in patients with GIST in a long-term study and to elucidate the relationships between clinicopathological features and IM C_min_.

**Methods:**

In 204 patients with intermediate- or high-risk GIST who were taking IM, IM C_min_ was analyzed. Patient data were grouped according to the duration of medication (A: 1–3 months, B: 4–6 months, C: 7–9 months, D: 10–12 months, E: ≤12 months, F: 12<–≤36 months, G: >36 months). The correlation between IM C_min_ at different time stages and clinicopathological characteristics was assessed.

**Results:**

Statistically significant differences were observed between Groups A, C, and D (*P* = 0.049 and 0.01, respectively). In Group E, IM C_min_ correlated with sex (*P* = 0.049) and age (*P* = 0.029) and negatively correlated with body weight, height, and body surface area (*P* = 0.007, 0.002, and 0.001, respectively). In Groups F and G, IM C_min_ was significantly higher in non-gastric operation patients than in patients with gastrectomy (*P* = 0.002, 0.036) and was significantly higher in patients with the primary sites of others than in the stomach (*P* < 0.001, = 0.012). In addition, IM C_min_ was much higher in patients with mutation sites other than KIT exon 11 in Group F (*P* = 0.011).

**Conclusion:**

This is the first study of IM C_min_ during the long-term treatment of patients with intermediate- or high-risk GIST. IM C_min_ was the highest for the first 3 months and then declined, and long-term administration of IM showed a relatively stable plasma trough level. The IM C_min_ correlated with different clinical characteristics at different durations of medication. This meant that future “trough level–clinicopathological characteristics” analyses should be time-point-specific. We also need to formulate time-specific medication monitoring plans in clinical practice to study disease progression caused by the occurrence of drug resistance.

## Introduction

Before the year 2000, there was no known effective therapy for gastrointestinal stromal tumors (GISTs). GIST treatment was developed on the basis of the finding that most GISTs have mutations in KIT and platelet-derived growth factor receptor-alpha (PDGFRA). These findings led to the development of effective systemic therapies in the form of small molecule tyrosine kinase inhibitors (TKIs), of which the prototype is imatinib (IM). It is a small molecule that inhibits intracellular autophosphorylation of tyrosine kinase receptors involved in GIST pathogenesis. After a decade of therapeutic use, IM is proven to be a highly effective targeted agent for the treatment of patients with advanced GIST, with a median overall survival of only approximately 19 months to approximately 5 years (1).

The success of these agents in advanced disease has prompted interest in their use as adjuvant treatment for patients at high risk of recurrence after the complete resection of a primary GIST tumor (2). Patients who received 3 years of IM had longer recurrence-free survival and overall survival times in the clinical trial SSGXVIII than those who received IM for 1 year (3). In the PERSIST-5 clinical trial, none of the patients with resected primary GIST who received 5 years of IM therapy with IM-sensitive mutations experienced disease recurrence during therapy (4). However, the optimal treatment duration remains unknown. We found that there was a tendency for prolonged IM adjuvant therapy in GIST patients with a high risk of recurrence.

In a retrospective study, a significantly shorter time to progression was observed in patients with IM trough levels below 1,100 ng/ml after 1 month of medication (5). A prospective pharmacokinetic study in patients with advanced GIST showed a significant decrease of approximately 30% in IM trough levels after long-term treatment. The authors of this study have called for conducting time-point-specific “trough level–clinical benefit” analyses in the future (6). IM plasma concentration changes over time in patients with advanced GIST. However, no similar research has been conducted on IM adjuvant therapy for patients with GIST. In this context, the primary aim of this long-term study is to assess the changes in IM plasma concentrations in patients with intermediate- or high-risk gastrointestinal stromal tumors. As IM plasma concentration may have changed over time, our secondary aim is to elucidate the factors affecting IM concentration in blood at different time points to formulate better time-specific medication monitoring plans in clinical practice.

## Methods

### Patient eligibility

This retrospective study was conducted at the First Affiliated Hospital of Chongqing Medical University. From April 2015 to December 2018, only patients with a primary GIST diagnosis who underwent surgery with curative intent (R0) were included in this study. The inclusion criteria were as follows: (1) pathologically confirmed GIST; (2) according to the guidelines and expert consensus (7, 8), patients were identified with an intermediate or high risk of relapse, with at least one of the following features: longest tumor diameter of >10.0 cm and mitotic count >10 mitoses per 50 high-power fields of the microscope, a tumor diameter of >5.0 cm and mitotic count >5 mitoses per 50 high-power fields of the microscope, a small intestinal tumor diameter >5.0 cm or mitotic count >5 mitoses per 50 high-power fields of the microscope, or tumor rupture before surgery or at surgery; (3) IM taken by patients was produced by Novartis (Switzerland); (4) patients with GIST took IM at a fixed 400 mg daily dose; and (5) good compliance (take IM regularly). The exclusion criteria were as follows: (1) serious comorbidity; (2) oral administration restricted because of significant gastrointestinal bleeding or obstruction; (3) treatment with drugs known to induce or inhibit CYP3A4 or *P*-glycoprotein and inhibit the human organic cation transporter 1 if no alternative medication was available, or if the patient was unwilling to change the medication.

### Sample collection and pharmacokinetic analysis

For quantifying IM plasma concentration, blood sampling was conducted at 24 ± 3 h following the previous dose of IM in patients with GIST. Patient data were grouped according to the duration of medication with IM (Group A: 1–3 months, Group B: 4–6 months, Group C: 7–9 months, Group D: 10–12 months, Group E: ≤12 months, Group F: 12<–≤36 months, Group G: >36 months). Methods for blood sample processing and storage, as well as IM quantitative analysis, have been previously described (9). The lower limit of quantification was set at 50 ng/ml.

### Data collection and correlation analysis

The patients regularly visited the same surgeon every 3–6 months. The clinical characteristics of all patients with GIST were recorded. These clinical characteristics included age, sex, gene mutation site, risk level, primary tumor site, CD34/CD117/DOG-1 (positive or negative), surgical procedures, stable disease or progression, liver metastasis, ki67, and the maximum diameter of the tumor. Body weight was measured at the time of blood sampling, and the body surface area was calculated. A retrospective analysis was conducted to assess the relative factors affecting IM plasma trough concentration (IM C_min_) after different durations of IM medication.

### Statistical analysis

All data are presented as mean ± standard deviation and were analyzed using SPSS 22.0. Differences in plasma concentrations between groups were compared using one-way ANOVA with the least significant difference or Dunnett's test. Correlations between IM trough plasma concentrations and continuous variables that fitted a normal distribution were analyzed by univariate linear regression (Pearson test); continuous variables that did not conform to normal distribution were analyzed using the Spearman rank correlation. *P* values <0.05 were considered statistically significant.

## Results

### Patient characteristics

**Figure 1:**
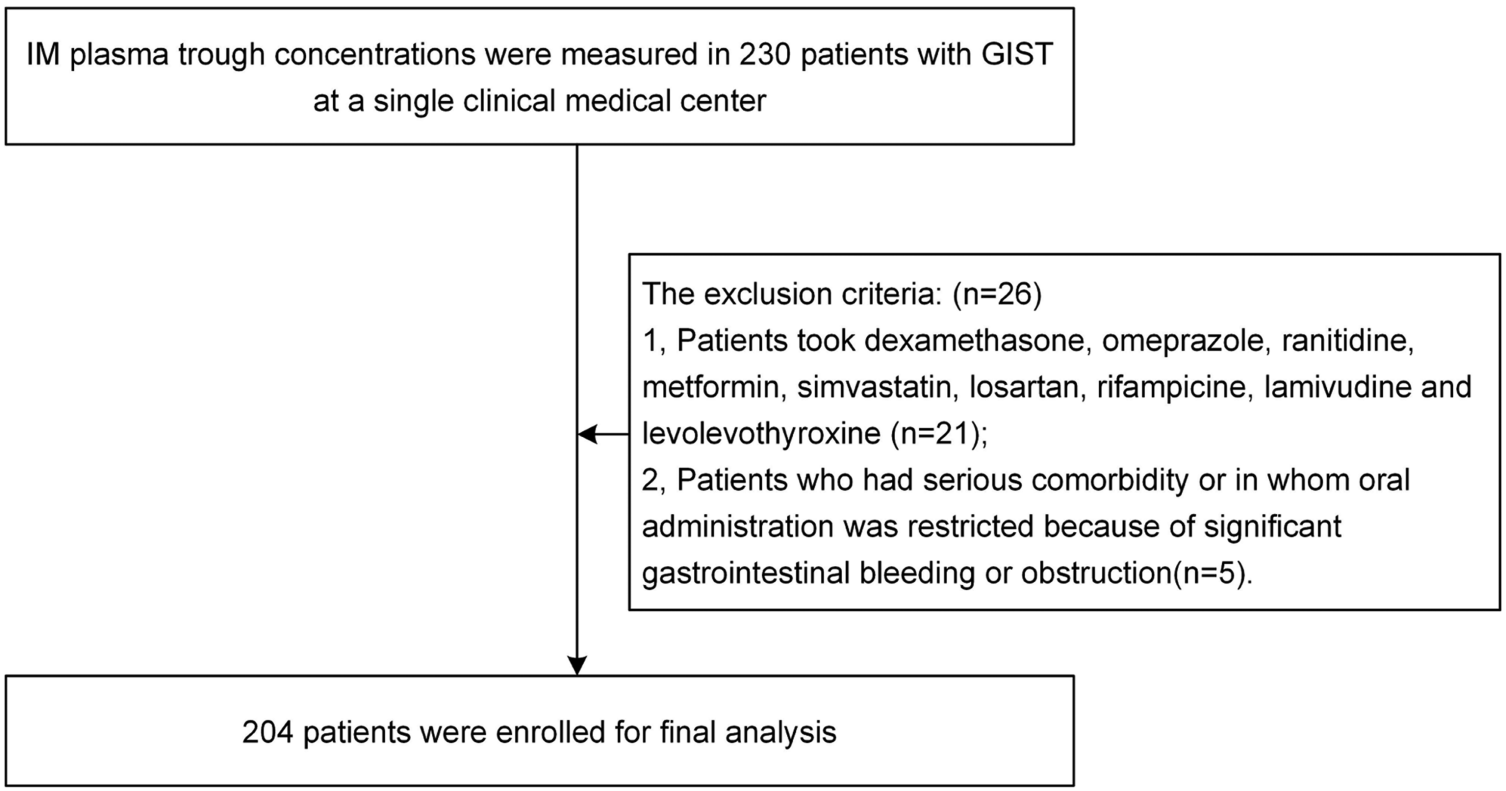
Flow chart of patient selection.

The IM plasma trough concentrations were measured in 230 patients with GIST. Twenty-one patients were excluded for dexamethasone, omeprazole, ranitidine, metformin, simvastatin, losartan, rifampicin, lamivudine, or levothyroxine. Five patients with serious comorbidities or in whom oral administration was restricted because of significant gastrointestinal bleeding or obstruction were excluded. Finally, 204 patients were included in the study. The details are provided in Figure 1. The median age was 58 years (range: 31–85 years), and the sex ratio was 1.1:1 (53.3% were female). The clinical features of all patients are listed in Table 1. Almost all patients experienced adverse reactions; these included periorbital edema, facial edema, diarrhea, vomiting, dyspepsia, and fatigue. According to the National Cancer Institute Common Terminology Criteria for Adverse Events, all patients included belonged to Grade 1, and the drug dose was not adjusted. None of the patients experienced serious or life-threatening adverse events.

**Table 1:** The clinical features of the patients enrolled in the study.

Characteristic	Included patients (*n* = 204)
**Age, years**	
Mean (±SD)	56.7 ± 11.2
Median (range)	56 (31-80)
**Gender, *n* (%)**	
Female	107 (52.5)
Male	97 (47.5)
**Primary tumor site, *n* (%)**	
Stomach	98 (48.0)
Small intestine	85 (41.7)
Other	21 (11.3)
**Primary tumor size, cm**	
Mean (±SD)	7.5 ± 4.3
Median (range)	6.2 (2.5-30)
**Mitotic count, *n* (%)**	
≤5	139 (68.1)
>5, ≤10	44 (21.6)
>10	21 (10.3)
**Mutation, *n* (%)**	
Kit Exon 11	112 (54.9)
Wild-type GIST	17 (8.3)
Other sites	18 (8.8)
Mutations not detected	57 (27.9)

### IM C_min_ among different medication durations

**Figure 2:**
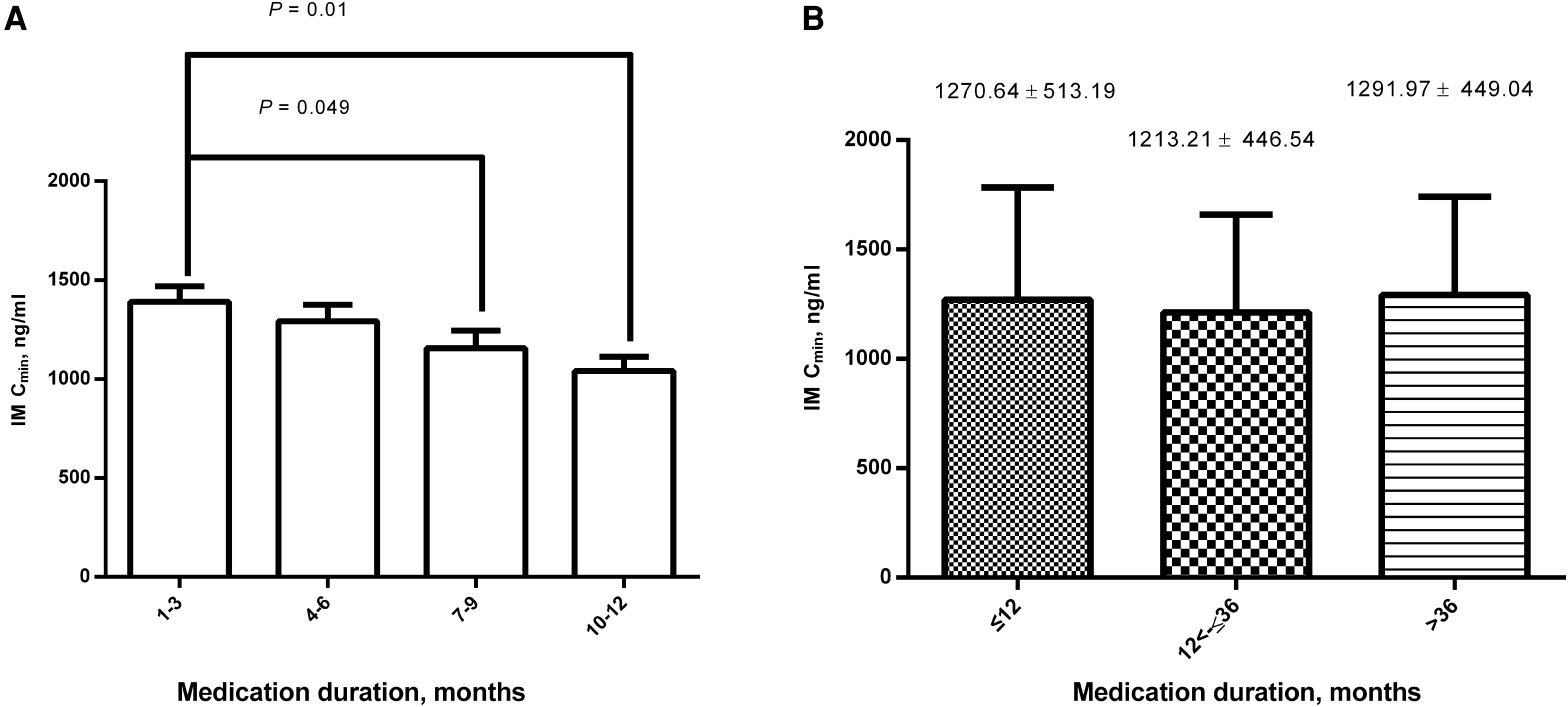
Comparison of IM C_min_ among different medication durations. (**A**) There were statistically significant differences between 1– 3 months, 7–9 months, and 10–12 months; (**B**) There were no statistically significant differences between ≤12, 12<–≤36, and >36 months. IM, imatinib; C_min_, plasma trough levels.

Group divisions according to the duration of medication with IM were as follows: Group A: 1–3 months, Group B: 4–6 months, Group C: 7–9 months, Group D: 10–12 months, Group E: ≤12 months, Group F: 12<–≤36 months, and Group G: >36 months. The IM C_min_ of each group were 1390.13 ± 553.80, 1292.94 ± 441.95, 1156.53 ± 450.24, 1040.01 ± 301.70, 1270.64 ± 513.19, 1213.21 ± 446.54, and 1291.97 ± 440.04 ng/ml, respectively. There were statistically significant differences between Groups A, C, and D (*P* = 0.049 and 0.01, respectively) (Figure 2A), but there were no statistically significant differences between Groups E, F, and G (F = 0.485, *P* = 0.617) (Figure 2B).

### Clinical characteristics and IM C_min_

**Figure 3:**
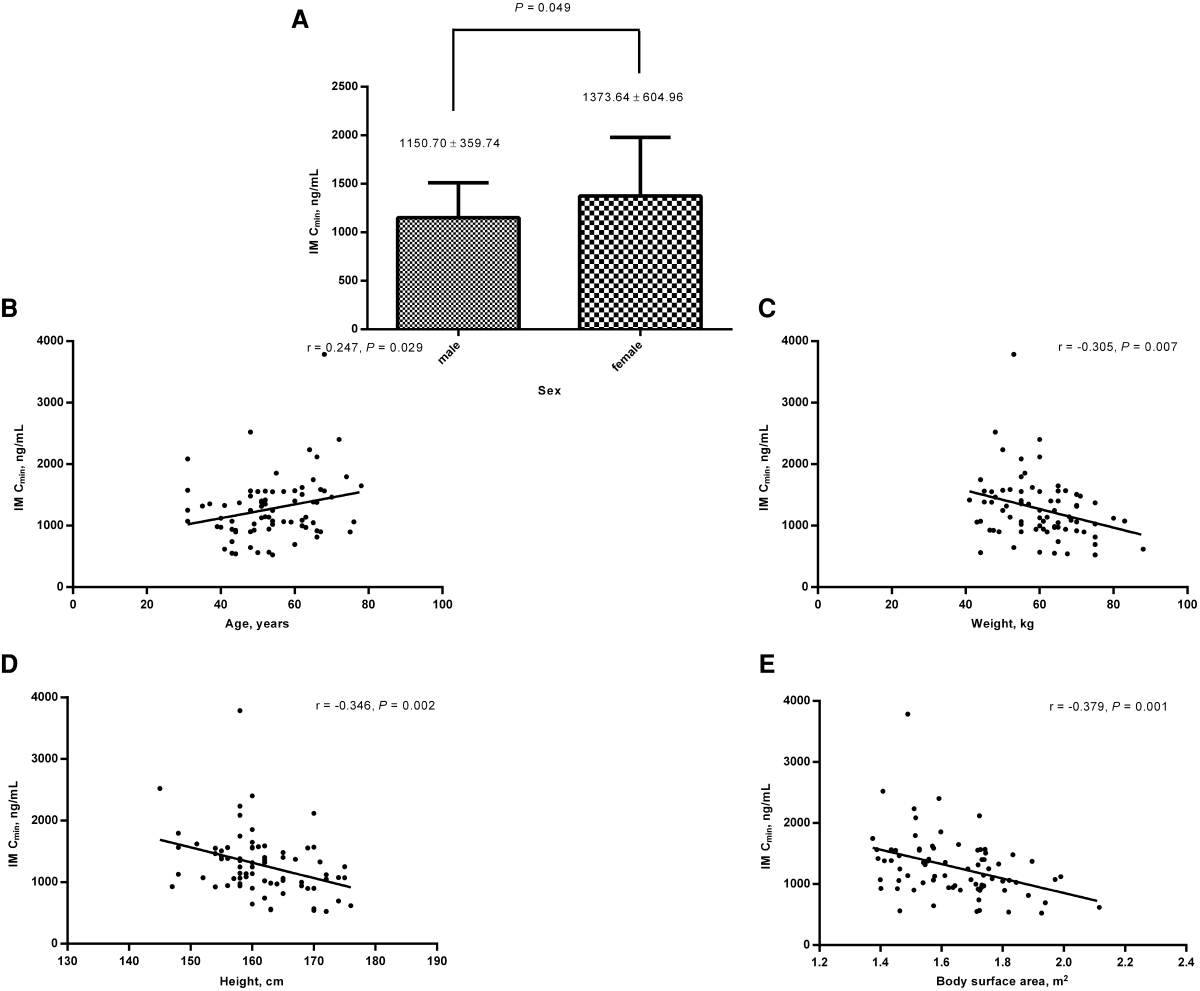
Relationships between clinical characteristics and IM C_min_ within the medication duration of 12 months. (**A**) IM C_min_ was significantly higher in female patients than in male patients; IM C_min_ correlated with age (**B**) and negatively correlated with body weight (**C**), height (**D**), and body surface area (**E**). IM, imatinib; C_min_, plasma trough levels.

Correlation analyses between the clinical characteristics and IM C_min_ were performed retrospectively. Within the medication duration of 12 months, IM C_min_ was significantly higher in female patients than in male patients (1,373.64 ± 604.96 ng/ml (*n* = 42) vs. 1,150.7 ± 359.74 ng/ml (*n* = 39), *P* = 0.049) (Figure 3A). IM C_min_ correlated with age (*r* = 0.247, *P* = 0.029) and negatively correlated with body weight, height, and body surface area (*r* = −0.305, −0.346, −0.379, *P* = 0.007, 0.002, and 0.001, respectively) (Figures 3B-E).

**Figure 4:**
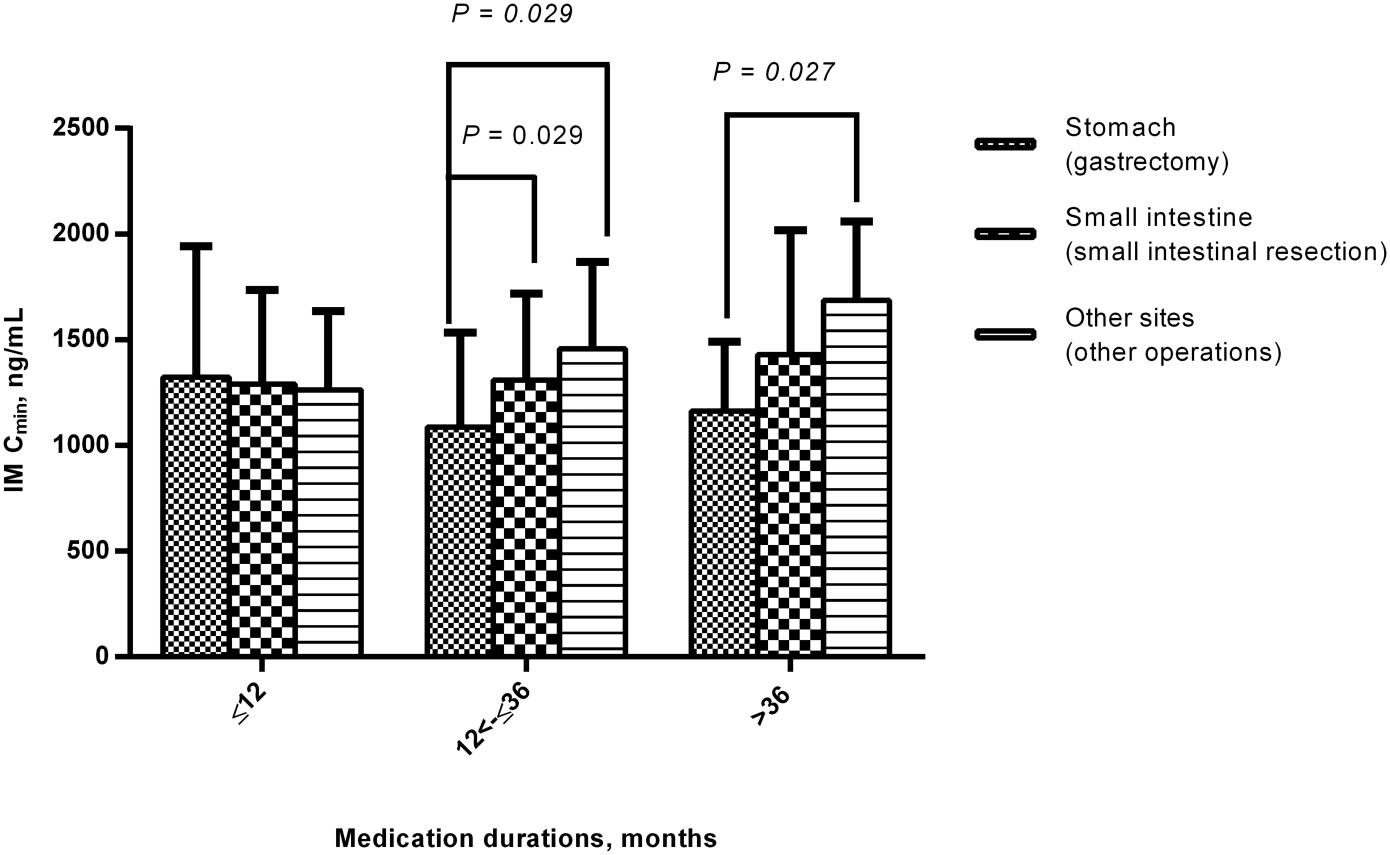
Comparison of IM C_min_ among different primary site or surgical procedure groups during the medication period. When the medication duration was 12<–≤36 months and >36 months, IM C_min_ was significantly higher in patients with the primary site of the small intestine (small intestinal resection) or other sites (other operations) than the stomach (gastrectomy). IM, imatinib; C_min_, plasma trough levels.

When the medication duration was 12<–≤36 months, IM C_min_ was significantly higher in patients with the primary site of the small intestine (small intestinal resection) and other sites (other operations) than the stomach (gastrectomy) (1,309.77 ± 407.12 ng/ml (*n* = 32) and 1,457.02 ± 410.39 ng/ml (*n* = 8) vs. 1,087.23 ± 452.74 ng/ml (*n* = 44), *P *= 0.029) (Figure 4). When the medication duration was >36 months, IM C_min_ was also significantly higher in patients with primary sites of the small intestine (small intestinal resection) or other sites (other operations) than the stomach (gastrectomy) (1,687.23 ± 372.34 ng/ml (*n* = 4) vs. 1,162.59 ± 327.67 ng/ml (*n* = 25), *P* = 0.027) (Figure 4).

**Figure 5:**
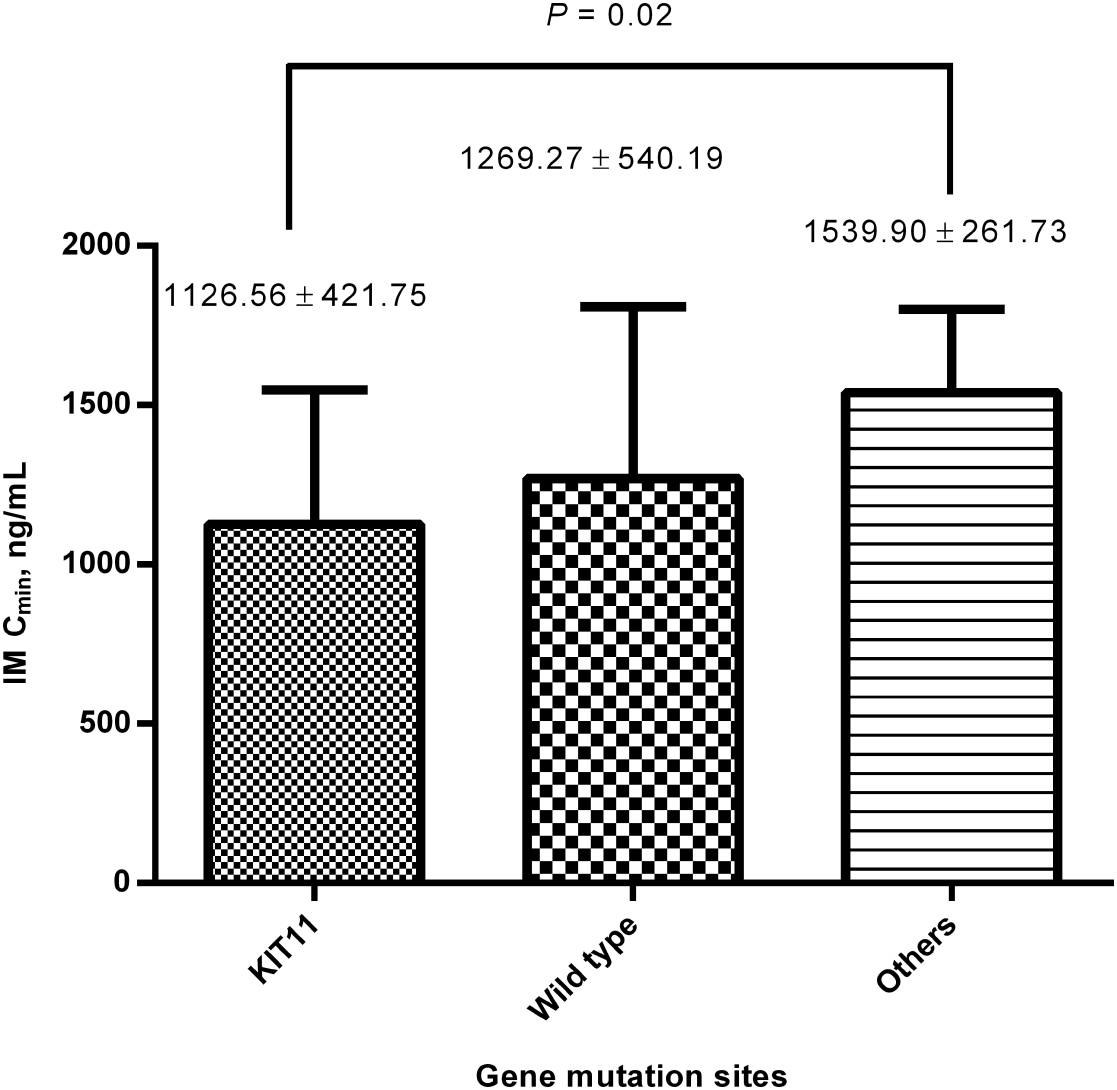
Comparison of IM C_min_ between different gene mutation sites within the medication for 12<–≤36 months. The IM C_min_ was much higher in patients with mutation sites other than KIT exon 11 mutations. IM, imatinib; C_min_, plasma trough level.

In addition, IM C_min_ was much higher in patients with mutation sites other than KIT exon 11 mutation during the medication duration of 12<–≤36 months (1,539.90 ± 261.73 ng/ml (*n* = 7) vs. 1,126.56 ± 421.75 ng/ml (*n* = 52), *P* = 0.02) (Figure 5).

## Discussion

This is the first study that focuses on the changes in IM C_min_ during long-term treatment in patients with adjuvant therapy for GIST. We have shown here that during the first year, IM C_min_ was highest for the first 3 months and then declined. There were no significant differences in the C_min_ of IM among patients who took the drug for 1 year, 1 to 3 years, and more than 3 years. We also analyzed the relationships between the clinicopathological characteristics of the patients and IM C_min_ at different time points. Within the first year of treatment, IM C_min_ correlated with age, sex, body weight, height, and body surface area. When the medication duration was 12–36 months, IM C_min_ correlated with the primary site of GIST, surgical procedures, and gene mutations. When the medication duration was over 36 months, IM C_min_ was significantly higher in patients with primary sites other than the stomach and the corresponding surgical procedures.

For advanced GIST, although IM treatment has proven efficacy, disease progression ultimately occurs because of drug resistance. An altered expression of these transporters may lead to increased clearance of the drug and lower plasma concentrations of IM over time. In a retrospective study, Demetri et al. reported that a significantly shorter time to progression was observed in patients, which showed 1-month IM trough levels below 1,100 ng/ml after 1 month of medication (5). In real-life settings, the concentration of IM significantly influences the duration of tumor control treatment in GIST patients, with a C_min_ threshold of 760 ng/ml associated with prolonged progression-free survival (10). The reason for the difference in threshold might be that blood sampling was conducted at different times, as Eechoute et al. found a significant decrease of approximately 30% in IM exposure after long-term treatment (6).

To the best of our knowledge, no similar study has been conducted on IM adjuvant therapy. But this is a crucial parameter to monitor IM C_min_ in populations with intermediate- or high-risk GIST for a long time. This is the first step in determining the C_min_ threshold value and therapeutic drug monitoring for IM in GIST after complete excision. In this study, during the first year of IM, the plasma trough level was highest for the first 3 months and then declined. During long-term treatment, the IM C_min_ did not decrease significantly but remained stable or slightly increased after 3 years, but the difference was not statistically significant. This result was consistent with those reported by Eechoute et al. and Yoo et al. in patients with advanced GIST (6, 11).

Exposure to IM during treatment may be influenced by various factors. Pharmacokinetic studies in patients with chronic myeloid leukemia and GIST have found that variables that were significantly influenced or associated with IM C_min_ included white blood cell count, age, body weight, body surface area, and previous gastrectomy (12–14). Our previous study found that gastrectomy, body weight, body surface area, and sex may impact IM C_min_ in the Chinese population (9). However, none of the studies focused on patients who received IM adjuvant treatment, and no subgroup analysis was performed by considering the time from the initiation of IM treatment. In this study, patients were grouped according to the duration of IM treatment. Although no statistically significant differences in IM C_min_ were found for different durations of medication, we were surprised to notice that IM C_min_ correlated with different clinical characteristics for different durations of medication.

Sex, age, body weight, height, and body surface area were the factors influencing IM C_min_ within 1 year of taking medicine. IM C_min_ was higher in females than in males. Some researchers believe that this difference may be attributed to differences in body weight between sexes (13). In addition, our research found that adherence was poorer in male patients than in female patients, which might also be the reason for the lower IM C_min_ (15). Whether the dose of the drug should be adjusted for body weight, body surface area, or sex remains a controversial issue. First of all, the results of this study need to be verified by using a larger sample size. Moreover, the influence of the above factors on blood concentration was not continuous but existed only at a certain stage of imatinib adjuvant therapy. However, these results could be useful during postoperative follow-up. For example, more attention should be paid to whether obese male patients experience tumor recurrence because of low IM C_min_.

When the medication duration was over 12 months, IM C_min_ was significantly lower both in patients with gastrectomies and in the primary site of the stomach. Although wedge gastric resection was the main type of operation in our study, low IM C_min_ was also associated with gastrectomy. Because IM tablets dissolved easily and rapidly at pH 5.5 or less (16), the reduced absorption of IM might be caused in part by a lack of gastric acid secretion in patients who had undergone gastric resection.

In addition, after excluding patients with wild-type GIST, IM C_min_ was found to be much higher in patients with mutation sites other than KIT exon 11 mutation during the medication duration of 12–36 months. KIT exon 11 mutation is the most significant benefit of GIST adjuvant therapy (17, 18), but the blood concentration of patients with KIT exon 11 mutation is not the highest. This suggests that the benefit to these patients might be mainly due to the biological behavior caused by mutation rather than the contribution of high blood IM concentration.

This study has several limitations. First, only 204 patients were included; the results of this study need to be verified by using a larger sample size. Second, there are several other factors influencing IM C_min,_ but this study does not clarify all of them. Finally, this is a retrospective study, and there is a possibility that selection bias could influence the outcomes, even though strict screening of the enrolled patients was carried out. In future research, we hope to further expand the sample size, undertake a multicenter study, and focus on patient survival.

## Conclusion

IM plasma trough levels during long-term treatment of patients with intermediate- or high-risk GIST showed that IM C_min_ was the highest for the first 3 months and then declined, and the long-term administration of IM showed a relatively stable C_min_. The IM C_min_ correlated with different clinical characteristics at different durations of medication. Our results remind us again that “trough level–clinicopathological characteristics” analyses should be time-point-specific in the future. Prospective studies with larger sample sizes are required to verify the results of this study. There is still a long way to go in adjusting the dosage of IM according to IM C_min_ and the influencing factors. However, in clinical practice, according to the rules found in this study, we can develop targeted drug monitoring plans for special populations to prevent disease recurrence caused by a low IM C_min_.

## Data Availability

The raw data supporting the conclusions of this article will be made available by the authors without undue reservation.
